# The Prevalence of Trichomoniasis in Women Referred to Clinical Centers in South of Tehran, Iran during 2015–2016

**Published:** 2018

**Authors:** Akram AZAMBAKHTIAR, Bahram NIKMANESH, Mostafa REZAEIAN, Nasrin DASHTI, Fatemeh SAFARI, Mitra ZAREBAVANI

**Affiliations:** 1. Dept. of Parasitology and Mycology, School of Public Health, Tehran University of Medical Sciences, Tehran, Iran; 2. Dept. of Medical Laboratory Sciences, School of Allied Medical Sciences, Tehran University of Medical Sciences, Tehran, Iran

**Keywords:** *Trichomonas vaginalis*, Prevalence, Risk factors, Iran

## Abstract

**Background:**

This study aimed to estimate the prevalence of trichomoniasis infection among females in Tehran, Iran.

**Methods:**

This study was conducted on 482 women referred to the 6 obstetrics and gynecology centers of Tehran during 2015–2016. Some information including education, occupation, and number of sexual partners was obtained and clinical signs and symptoms of the genital tract were diagnosed by clinical examination. Two swabs were collected from the posterior fornix of patients. Two laboratory techniques, wet mount, and culture were carried out. Finally, statistical analysis test was performed using SPSS software version 16.0.

**Results:**

Age distribution of patients was 15–60 yr. *Trichomonas vaginalis* was detected in 2 out of 482 participants (0.41%). All of the infected individuals were married (0.43%) and they had unique sexual partner and all of them had clinical symptoms. Significant association was observed between incidence of *T. vaginalis* infection and educational levels (*P*= 0.03), occupation (*P*=0.006), clinical symptoms (*P*=0.001), marriage (*P*=0.006) and bacterial infection (*P*=0.018).

**Conclusion:**

The prevalence of trichomoniasis was low and its incidence was associated with several risk factors.

## Introduction

Nowadays, *Trichomonas vaginalis*, *Chlamydia trachomatis*, *Neisseria gonorrhoeae*, are responsible for over 351.7 million infections annually. Although these infections can be treated, they can cause long-term complications in women and men. Trichomoniasis is the most prevalent remediable sexually transmitted infection (STI) in the world that related to the low socioeconomic situations. *T. vaginalis* can cause some serious complications for women, such as adverse pregnancy outcomes that appear by preterm rupture of membranes, preterm delivery, low-birth-weight infants, infertility and cervical cancer (1, 2).

Moreover, it can increase the human immunodeficiency virus (HIV) transmission (3). Much information remains regarding *T. vaginalis* epidemiology (4). The incidence of *T. vaginalis* infection among women is associated with some risk factors like multi partners, non-stable partners), current or previous infections with other STIs such as Hsv2, HIV and syphilis (5, 6), sex work, drug use and incarceration (5). Many studies have found a positive correlation between *T. vaginalis* infection and age in women, while in other studies this correlation was negative (7). The prevalence of *T. vaginalis* in men is far lower than women while the incidence of it is similar in men and women (4). In another sexually transmitted infection such as *Gonorrhea* the prevalence varies with trichomoniasis, in the trichomoniasis prevalence depends on the region, time studied and population (8). The prevalence estimation varies in different populations, but it has common ranges from 5% to 74% in women and 5% to 29% in men (9). Detection of sensitive diagnostic methods has been modified with some more sensitive diagnostic tools, especially polymerase chain reaction (PCR). Among the available methods, at least two methods are better for diagnosis *T. vaginalis*, for example, culture and wet mount microscopy (10).

In Iran, various studies have shown the prevalence of trichomoniasis between 2% to 8%. Furthermore, with respect to the cultural and social status, it can be reached by over 30% (11, 12). Recently, trichomoniasis is considered as one of the most common sexually transmitted diseases. This study aimed to estimate the prevalence of *T. vaginalis* infection among females in Tehran, Iran.

## Materials and Methods

This cross-sectional study was conducted in women referred to the 6 obstetrics and gynecology centers of Tehran, Iran during 2015–2016. A total of 482 women were randomly selected.

Inform written consent was obtained from all enrolled patients prior to the study and the study was approved by the university.

Patients were interviewed regarding demographic information such as age, education, occupation, a number of sexual partners, and clinical signs and symptoms of the genital tract were diagnosed by clinical examination. Two swabs were collected from the posterior fornix of patients and two laboratory techniques, namely, wet mount and culture were carried out for diagnosis of Trichomoniasis. A wet smear was performed immediately as a routine diagnostic procedure for a motile parasite, using the first swab. The second swab was for the Dorset culture. The culture was transferred to the School of Public Health in Tehran University of Medical Sciences and incubated at 37 °C for 24 h. Afterward, a fresh smear was prepared and examined microscopically for the presence of *T. vaginalis* daily, up to seven days.

Finally, statistical analysis test was conducted using SPSS software (ver. 16.0, Chicago, IL, USA). All results were expressed as mean ± standard deviation (SD), categorical data were presented as percentages and *P*-value<0.05 was considered significant.

## Results

Overall, 482 patients were enrolled in our study with age range of 15–60 yr. The age distribution was different, that 1.7% of them were <20, 25.5% were between 20 and 30, 46.3% between 31 and 40, 23% between 41 and 50 and 3.5% were between 51 and 60 yr. Moreover, 10.6% of subjects were illiterate, 52% had low diploma, 24.7% had a diploma and 12.7% had university education. Most of them were housewives (95.8%), while 4.2% were employed. Among them, 85% had no abortion, while 10.6%, 3.7%, and 0.7% had one, two and three abortions respectively. Most of the women were married (95.8%), 1.9% and 1.5% were single and divorced respectively. Moreover, of 482 subjects, 2.9% had a pregnancy, while 97.1% had not any pregnancy. The findings of contraceptive method showed that 7.9%, 4.8%, 5% and 0.8% of the subjects used condom, tablet, IUD, and ampoule respectively, 73% prevented naturally, 0.4% and 8.1% have a vasectomy and tubectomy, respectively.

*T. vaginalis* was detected in 2 out of 482 participants (0.4%) by wet mount technique, and by culture, only 1 case (0.2%) was *T. vaginalis* positive. All of the infected individuals were married (0.43%) and they had a unique sexual partner. In addition, 0.21% of the housewife women (1/462), and 5% of the employment women (1/20) were *T. vaginalis* positive. All of the infected patients had clinical symptoms such as itching, dysuria, discharge malodorous and abdominal pain. 0.84% of the infected women had moderate WBC and 0.6% had many WBC, 1.01% were positive for bacteria and 0.26% were bacteria negative. 0.22% (1/450) of the women who did not use metronidazole was *T. vaginalis* positive and among women who used metronidazole, 3.12% (1/32) was *T. vaginalis* positive.

By performing Chi-squared test, significant association was observed between incidence of *T. vaginalis* infection and education levels of patients (*P*=0.03), job (*P*=0.006), clinical symptoms (*P*=0.001), marriage (*P*=0.006), bacterial infection (*P*=0.018), WBC (*P*=0.036) and consumption of metronidazole (*P*=0.002).

Potential risk factors for prevalent of *T. vaginalis* are shown in Table, 1, 2 and 3.

**Table 1:** The relationship between incidence of *T. vaginalis* and age, education and job

***Risk factors***		***Number (%)***	***T. vaginalis Positive***	***Total***
Age (yr)	20–30	123(25.5)	1(81)	482
	31–40	223(46.3)	1(0.44)	482
Education	L diploma	251(52)	1(0.39)	482
	University	61(12.7)	1(1.63)	482
Job	Housewives	462(95.8)	1(0.21)	482
	Employed	20(4.2)	1(5)	482

**Table 2:** The relationship between incidence of *T. vaginalis* and abortion, marriage, pregnant and contraceptive method

***Risk factors***		***Number (%)***	***T. vaginalis Positive***	***Total***
Abortion	Without abortion	410(85)	1(0.24)	482
	One abortion	51(10.6)	1(1.96)	482
Marriage and pregnant	Married	462(95.8)	2(0.43)	482
	Non-pregnant	468(97.1)	2(0.42)	482
Contraceptive method	Prevented (natural)	352(73)	1(0.28)	482
	Prevented (tubectomy)	39(8.1)	1(2.56)	482

**Table 3:** The relationship between *T. vaginalis* and microscopic findings of wet smear

***Risk factors***	***Number (%)***	***T. vaginalis Positive***	***Total***
WBC (moderate)	119(24.7)	1(0.84)	482
WBC (many)	165(34.2)	1(60)	482
RBC	472(97.9)	2(0.42)	482
Epithelial (moderate)	132(27.4)	1(0.75)	482
Epithelial (many)	266(55.2)	1(0.37)	482
Bacteria-negative	383(79.5)	1(0.26)	482
Bacteria-positive	99(20.5%)	1(1.01)	482
Fungi-negative	442(92.1)	2(0.45)	482

## Discussion

Trichomoniasis is one of the most common STIs in the world with 248 million new cases (13). “In Islamic countries, some information regarding the prevalence of *T. vaginalis* infection was given from 1.2% in Libya, 2.1% in India, 3.2% in Jordan to 28.1% in Saudi Arabia” (10).

The epidemiology of *T. vaginalis* is not well known. Among high-risk women, the rates vary from 5% among female sex workers (FSW) in Pakistan (14) 9.5%, in Zimbabwe (15) to 53% among imprisoned women in the USA (16).

The frequency of trichomoniasis infection in this present study was lower than the several previous studies in Iran, as in Rafsanjan, south-central Iran, frequency of *T. vaginalis* in 162 symptomatic pregnant women, was 27% and 56.2% by using wet mount and culture methods, respectively (17). In Kashan city, Iran, the overall prevalence of *T. vaginalis* was 2% among 970 women and 235 men (10). The prevalence rates of 2.1% and 1.5% using the culture and wet mount methods, respectively in 600 women were attended the obstetric clinics in Kermanshah (18). In Shiraz, the prevalence of this infection was 11.2% among vulnerable household women (19). The frequency rates in non-pregnant women referred to health centers were 9.2% in Tabriz (10), 4.8% in Zanjan (18) and 3.2% in Tehran (11).

The frequency of *T. vaginalis* in less educated women was higher than the other groups (18). Moreover, the results from our study showed a significant association between the incidence of trichomoniasis and educational level in our study population (*P*<0.05). In this study, all of the infected women had clinical symptoms and by using statistical test, we observed the significant association between the incidence of *T. vaginalis* and presence of symptoms (*P*<0.05), that these results were according to the studies conducted in Kermanshah city (18) and Kashan city (10). While the study conducted in the USA, there was no correlation between clinical symptoms and positivity of infection (20). However, the presence of clinical symptoms can support the diagnosis of parasite, but it cannot be used alone to diagnosis the *T. vaginalis.*

The normal vaginal flora may be changed due to the presence of *T. vaginalis* infection which can cause bacterial vaginosis (21). Moreover, a longitudinal study showed the presence of bacterial vaginosis significantly increases the risk of acquiring *T. vaginalis* infection (22), that these findings were according to our study and a significant association between *T. vaginalis* and bacterial infection was observed (*P*<0.05).

In our study, all of those infected women were married and were housewives, which these results were consistent with the findings from other previous studies (10, 18). Moreover, we did not observe any association between abortion and trichomoniasis, like the study conducted in Khuzestan (23).

Therefore, the incidence of trichomoniasis is different in various communities, which may be due to different factors, including sociodemographic characteristics and hygiene behaviors and diagnostic methods used (17, 22). In this study, *T. vaginalis* was detected only in 2 out of 482 participants (0.4%) by wet mount technique.

## Conclusion

The prevalence of *T.vaginalis* infection was low and its incidence was associated with several risk factors such as educational level, occupation, marriage and bacterial infection. Further studies must be conducted among HIV, HBV, HCV, HPV positive women, immigrant men and women from other countries such as Iraq, Lebanon, Syria, Afghanistan, Pakistan, addicted women and men, beggars, women offenders in prison and other high-risk behaviors.
